# Expression and knockdown of zebrafish folliculin suggests requirement for embryonic brain morphogenesis

**DOI:** 10.1186/s12861-016-0119-8

**Published:** 2016-07-08

**Authors:** Emma J. Kenyon, Monique N. H. Luijten, Harmeet Gill, Nan Li, Matthew Rawlings, James C. Bull, Yavor Hadzhiev, Maurice A. M. van Steensel, Eamonn Maher, Ferenc Mueller

**Affiliations:** School of Clinical and Experimental Medicine, College of Medical and Dental Sciences, University of Birmingham, Edgbaston, Birmingham, B15 2TT UK; Department of Dermatology and GROW School for Oncology and Developmental Biology, Maastricht University Medical Center, Maastricht, The Netherlands; Department of Biosciences, College of Science, Swansea University, Swansea, SA2 8PP Wales UK; Sussex Neuroscience, School of Life Sciences, University of Sussex, Falmer, Brighton, UK; School of Medicine and School of Life Sciences, University of Dundee, Dow Street, Dundee, UK; Institute of Medical Biology, Immunos, 8A Biomedical Grove, Singapore, Singapore

**Keywords:** Birt-Hogg-Dubé syndrome (BHD), Zebrafish, *flcn*, Folliculin, zFucci, Cell cycle, Brain

## Abstract

**Background:**

Birt-Hogg-Dubé syndrome (BHD) is a dominantly inherited familial cancer syndrome characterised by the development of benign skin fibrofolliculomas, multiple lung and kidney cysts, spontaneous pneumothorax and susceptibility to renal cell carcinoma. BHD is caused by mutations in the gene encoding Folliculin (FLCN). Little is known about what FLCN does in a healthy individual and how best to treat those with BHD. As a first approach to developing a vertebrate model for BHD we aimed to identify the temporal and spatial expression of *flcn* transcripts in the developing zebrafish embryo. To gain insights into the function of *flcn* in a whole organism system we generated a loss of function model of *flcn* by the use of morpholino knockdown in zebrafish.

**Results:**

*flcn* is expressed broadly and upregulated in the fin bud, somites, eye and proliferative regions of the brain of the Long-pec stage zebrafish embryos. Together with knockdown phenotypes, expression analysis suggest involvement of *flcn* in zebrafish embryonic brain development. We have utilised the zFucci system, an *in vivo,* whole organism cell cycle assay to study the potential role of *flcn* in brain development. We found that at the 18 somite stage there was a significant drop in cells in the S-M phase of the cell cycle in *flcn* morpholino injected embryos with a corresponding increase of cells in the G1 phase. This was particularly evident in the brain, retina and somites of the embryo. Timelapse analysis of the head region of *flcn* morpholino injected and mismatch control embryos shows the temporal dynamics of cell cycle misregulation during development.

**Conclusions:**

In conclusion we show that zebrafish *flcn* is expressed in a non-uniform manner and is likely required for the maintenance of correct cell cycle regulation during embryonic development. We demonstrate the utilisation of the zFucci system in testing the role of *flcn* in cell proliferation and suggest a function for *flcn* in regulating cell proliferation in vertebrate embryonic brain development.

**Electronic supplementary material:**

The online version of this article (doi:10.1186/s12861-016-0119-8) contains supplementary material, which is available to authorized users.

## Background

Birt-Hogg-Dubé syndrome (BHD) is a dominantly inherited familial cancer syndrome characterised by the development of benign skin fibrofolliculomas, multiple lung and kidney cysts, spontaneous pneumothorax and susceptibility to renal cell carcinoma [1, 2]. The syndrome results from inactivating mutations in the gene encoding folliculin (FLCN) [3, 4] which has been suggested to be a *rab35* specific Guanine Nucleotide Exchange Factor (GEF) [5]. BHD patients only show a mutation in one copy of the *FLCN* gene [6], which suggests that several BHD symptoms may be due to abnormal levels of FLCN rather than its complete loss. This could explain why expression of mutant FLCN has been seen in a BHD-associated renal carcinoma [7]. FLCN has been implicated in the regulation of various signalling pathways and cellular processes including cellular metabolism through mTOR, AMPK and HIF1α, transcriptional regulation, JAK-STAT signalling, cell adhesion, ciliogenesis, lysosomal biogenesis and autophagy [7–17]. However, several groups have generated conflicting data on the consequences of FLCN deficiency and how these lead to the clinical manifestations associated with BHD is not clear. This leads to the question of what FLCN does in a healthy individual and how best to treat those with BHD.

Towards answering this question investigations in a whole organism model system provide important insights into the nature and evolutionary conservation of BHD-related effects on target signalling pathways. As homozygous mutations of *Flcn* is early embryonic lethal in mice, rats and dogs [18] , the utilisation of alternative animal models may be more informative about the developmental functions of Flcn. The external development of zebrafish make it a valuable vertebrate system, in which to elucidate both the function of *flcn* and the molecular pathways of oncogenesis [19].

Here we study the role of *flcn* in zebrafish development using morpholino oligonucleotides to generate a zebrafish loss of function (LOF) BHD model and we hypothesise that this could provide insights into the biological functions of Folliculin. We aim to identify the temporal and spatial expression of *flcn* transcripts in the developing embryo and reconcile this with the phenotype associated with the morpholino knock-down of zebrafish *flcn* to gain an insight into what *flcn* may do in the developing embryo.

## Results

### Isolation and expression of the zebrafish flcn gene

**Fig. 1:**
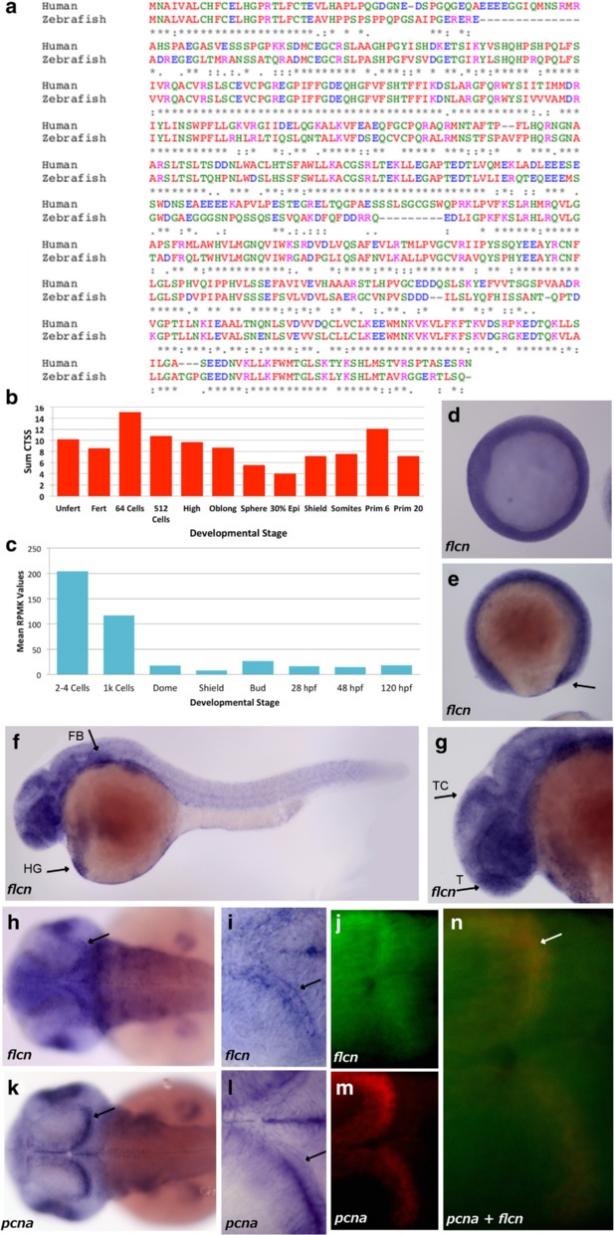
Conservation and expression of zebrafish *flcn*. **a** Comparative alignment of human *flcn* and zebrafish *flcn* protein sequence using Clustal Omega. (*) indicates positions which have a single, fully conserved residue, (:) indicates conservation between groups of strongly similar properties (.) indicates conservation between groups of weakly similar properties, Red = Small (small + hydrophobic), Blue = Acidic, Magenta = Basic – H, Green = Hydroxyl + sulfhydryl + amine + G and Grey = Unusual amino/imino acids. **b** Graph showing CAGE CT values for the zebrafish *flcn* gene over time, where CT values are the sum of all CAGE detected Transcription Start Site (CTSS) values , representing the number of cage tags (initiation instances) occurring at the same base (normalized tags per million (tps)) in the *flcn* promoter region. The count value was normalized according to total mapped tags and CTSS instances. **c** Graph showing the RNAseq RPKM values for zebrafish *flcn* over time, where the RPKM values represent the mean of the signal coverage value in the promoter region, therefore the sum of the total signal in the whole gene locus region divided by the number of bases with coverage. **d** Shield stage embryo showing ubiquitous expression. **e** 4 somite stage embryo showing low level expression over the whole embryo with increased expression seen in the region of the tail bud (*arrow*). **f** Prim-10 stage embryo showing low level expression in the whole embryo with more pronounced expression in the forebrain and hindbrain areas, hatching gland (HG, *arrow*) and the fin bud (FB, *arrow*). **g** Head of Prim-10 stage embryo showing expression in the tectum (TC) and telencephalon (T) (*arrows*). **h** Long-pec stage embryos showing expression in the brain (*arrow*) and retina of the embryo. **i** Long-pec stage embryo showing pronounced expression in the posterior tectum of the brain (*arrow*). **j** Head region of a Long-pec stage embryo showing expression of *flcn* (*green*) in the posterior tectum. **k** Long-pec stage embryo showing *pcna* expression in the retina and brain (*arrow*). **l** *pcna* expression in the posterior tectum of a Long-pec stage embryo (*arrow*) (**m**) head region of the same Long-pec stage embryo as panel J showing *pcna* (*red*) in the posterior tectum (**n**) head region of a Long-pec stage embryo showing expression of both *pcna* (*red*) and *flcn* (*green*) with co-localisation (*yellow*) in the posterior tectum (*n* = 20 embryos for all WMISH)

Based on a sequence search in the zebrafish genome, we identified *flcn* (ENSDARG00000062385), a FLCN homolog with very high sequence similarity to the human gene (ENSG00000154803) (Fig. 1a). To address when and where *flcn* is expressed we utilized our published [20] genome level analysis of the zebrafish transcriptome (by RNA Sequencing (RNASeq) and CAGE sequencing) and extracted *flcn* expression information at various stages of zebrafish development. Zebrafish *flcn* is expressed maternally, both in the unfertilized egg and in the pre MBT embryo. flcn is transcribed by the embryo during all stages of development at least up to larval stages. (Fig. 1b and c). We cloned a full open reading frame of the zebrafish *flcn* gene product by RT-PCR from protruding-mouth stage zebrafish larvae and verified the integrity of the sequence by sequencing and alignment to the zebrafish genome. To identify the *flcn* expression dynamics during embryo development we conducted whole mount *in situ* hybridization at several stages. At the start of gastrulation *flcn* expression can be seen at a low level throughout the whole embryo (Fig. 1d) and this expression remains present at subsequent developmental stages. In addition at four somite stage there is increased expression in the region of the tail bud (Fig. 1e*arrow*) while at Prim-5 stage there is increased expression in the myotome border/somatic furrow, eye, hatching gland (Fig. 1f*arrow* HG), fin bud (Fig. 1f*arrow* FB) tectum (Fig. 1g*arrow* T) and telencephalon (Fig.1g*arrow* TC). At long-pec stage pronounced expression is detected in a thin layer of cells in the posterior tectum (Fig. 1h*arrow*, i and j) and the fin (Fig. 1h). This expression is compared to that of the *proliferating cell nuclear antigen* (*pcna*) gene, using whole mount *in situ* hybridization (Fig. 1k, l and m) where it can be seen that there is an overlap in the expression of *pcna* and *flcn* in posterior tectum. The overlapping expression in the posterior tectum can be confirmed by fluorescent double *in situ* hybridization of *flcn* (Fig. 1j and n) and *pcna* (Fig. 1m and n). These results suggest that *flcn* may play a role in the regulation of the proliferation of cells in the embryo particularly in the proliferative zone of the brain.

### Morpholino knockdown indicates embryonic functions of folliculin

**Fig. 2:**
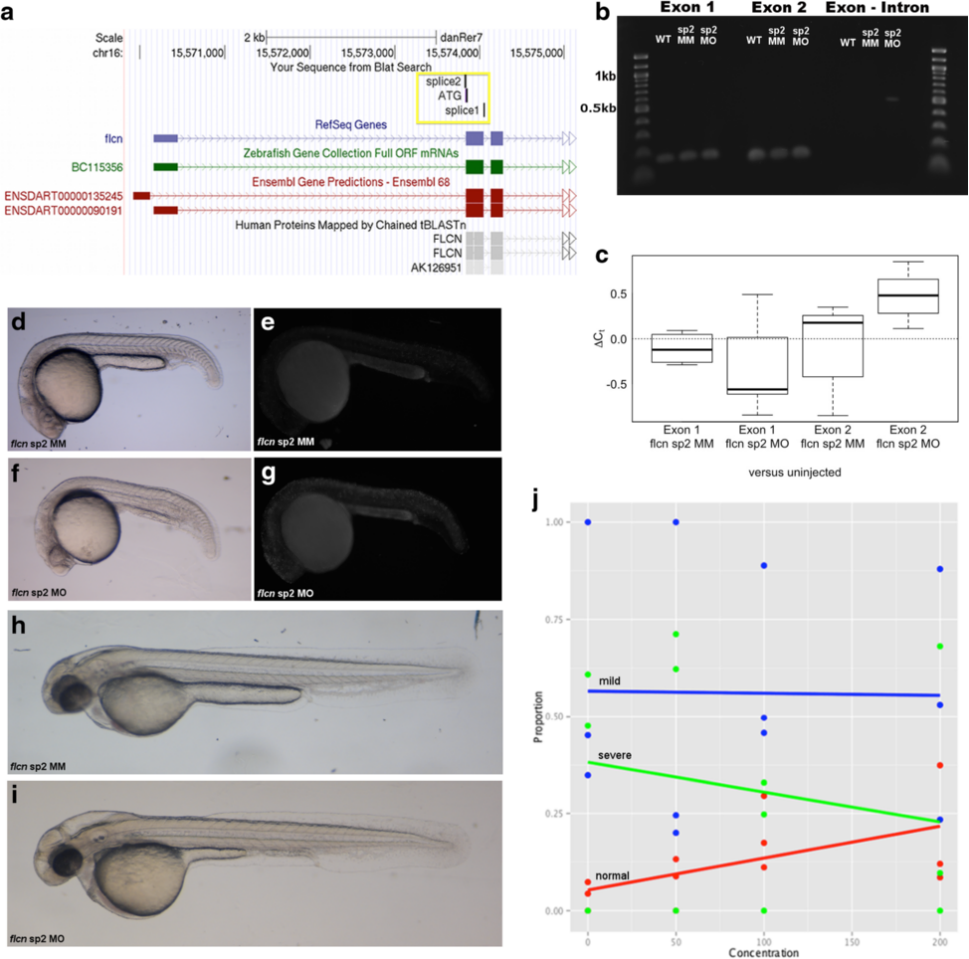
Knockdown of *flcn* in zebrafish embryos. **a** Schematic from the UCSC genome browser showing the binding sites of the ATG and splice site morpholinos used to knockdown the *flcn* gene (*yellow box*) (**b**) Electrophoresis gel showing; amplified *flcn* exon 1 and *flcn* exon 2 transcripts in wild type (WT), 100 μM of mismatch morpholino injected (sp2 MM) and 100 μM of *flcn* splice 2 morpholino injected (sp2 MO) embryos and; amplified *flcn* exon 1 to intron transcripts in 100 μM of *flcn* splice 2 morpholino injected embryos (sp2 MO) with no transcript in wild type (WT) and 100 μM of mismatch morpholino injected (sp2 MM) embryos (**c**) Box-whisker plots of *flcn* qPCR. Results, ΔC_t_, indicate expression relative to uninjected embryos. Boxes denote interquartile range with central horizontal line showing median expression. Whiskers extend to 1.5 x interquartile range. MO = morpholino, MM = mismatch. **d** Prim-5 stage 100 μM control splice 2 mismatch morpholino injected embryo. **e** Prim-5 stage 100 μM control splice 2 mismatch morpholino injected embryo stained with Acridine Orange **f** Prim-5 stage 100 μM *flcn* splice 2 morpholino injected embryo (**g**) Prim-5 stage 100 μM *flcn* splice 2 morpholino injected embryo stained with Acridine Orange (**h**) Long-pec stage 100 μM control splice 2 mismatch morpholino injected embryo. **i** Long-pec stage 100 μM *flcn* splice 2 morpholino injected embryo. **j** Proportion of embryos that are normal (*Red*), show mild hydrocephalus, larger yolk and thinner yolk extension (*blue*) or severe hydrocephalus, enlarged yolk and very thin yolk extension (*green*) when injected with combinations of 100 μM *flcn* splice 2 morpholino and 50 pg/μl, 100 pg/μl or 200 pg/μl of *flcn* RNA. With increasing concentrations of RNA, there was as significant decrease in the proportion of severe hydrocephalus and corresponding increase in the proportion of normal phenotype (ordinal logistic regression, *n* = 3 where n is the number of independent injections at each concentration and *p* = 0.027)

To gain insight into the potential role of *flcn* in cell proliferation in the brain, as suggested by the elevated expression in the proliferative zones and to interrogate *flcn* function in general we applied morpholino antisense oligonucleotides (MO). These were designed to block either the translation start site or the mRNA splicing acceptor site of *flcn.* The latter is expected to lead to a retention of an intron and either nonsense mediated decay or a premature stop of translation and thus to knockdown Flcn protein expression (Fig. 2a)*.* Efficiency of morpholino-induced altered splicing of pre-mRNA and thus gene knockdown was assessed by RT-PCR and qPCR on total RNA extracted from a pool of injected embryos. In embryos injected with *flcn* splice 1 morpholino the RT-PCR product was larger than that seen in the mismatch control morpholino injected embryos indicating that the 88 bp intron was retained (Additional file 1: Figure S1A). Retention of this intron leads to a premature stop and thus knockdown of the Flcn protein is expected. We are currently unable to assess protein levels as attempts to use mammalian anti-FLCN as well as newly generated zebrafish Flcn antibodies failed to specifically recognise zebrafish Flcn protein in Western blots on embryo extracts (data not shown). In embryos injected with 100 μM of *flcn* splice 2 morpholino a product was seen when a forward primer in exon 1 and a reverse primer in intron 1 were used. No product was seen in uninjected embryos or those injected with 100 μM of mismatch control morpholino (Fig. 2b). qPCR using primer pairs in exon 1 and primer pairs in exon 2 shows a reduction in exon 2 transcripts in 100 μM of *flcn* splice 2 morpholino injected embryos when compared with 100 μM mismatch control morpholino injected or uninjected embryos (Fig. 2c). Expression from embryos injected with 100 μM of *flcn* exon 2 morpholino was significantly down regulated (higher Ct, Fig. 2c) compared with uninjected embryos (ΔC_t_ = 0.758, SE = 0.291, *t* = 2.61, *p* = 0.015.). Neither expression from *flcn* exon 2 mismatch morpholino-injected embryos (*t* = 0.141, *p* = 0.889), nor *flcn* exon 1 morpholino-injected embryos (MO, *t* = 1.30, *p* = 0.206 and MM, *t* = 0.407, *p* = 0.688) was significantly different from uninjected embryos.

Knockdown of the zebrafish *flcn* gene by microinjection of either 100 μM of splice 2 (Fig. 2f, g and i), 100 μM of splice 1 (Additional file 1: Figure S1C) or 600 μM of ATG- targeting (Additional file 1: Figure S1E) *flcn* morpholino into wild-type zebrafish embryos resulted in a clear and reproducible phenotype (Additional file 2: Table S1) while control injected embryo appeared unaffected. At 18 somite stage embryos injected with *flcn* morpholino appeared to show developmental arrest when compared with mismatch controls. At Prim-5 stage experimental embryos showed a 2 h delay in development, with U-shaped somites and non-linear notochord in the tail (Fig. 2f) and cell death in the brain and trunk as implied by an increase in Acridine Orange staining (Fig. 2g), when compared to mismatch control morpholino injected embryos (Fig. 2d and e). At Long-pec stage the phenotype was characterised by brain oedema and problems with tail circulation as well as larger yolk and thinner yolk extension (Fig. 2i, Additional file 1: Figure S1D and S1F) when compared to mismatch controls (Fig. 2h and Additional file 1: Figure S1C and S1E) which were indistinguishable from the uninjected embryos.

To confirm that the morphant phenotype is caused by reduced gene expression of *flcn*, we aimed to perform rescue experiments by injection of the *flcn* splice 2 morpholino and full-length *flcn* RNA. Therefore, the full open reading frame of zebrafish *flcn* was cloned into the pCS2+ vector and RNA was produced *in vitro*. As injection of *flcn* RNA into mismatch morpholino injected embryos resulted in an increased number of embryos showing cyclopia, heart oedema, circulatory defects and reduced axis, rescue of MO knockdown was assessed by abolishment of the hydrocephalus and yolk phenotype. Increasing the amount of *flcn* RNA in embryos injected with *flcn* splice 2 morpholino resulted in an increase in the number of embryos that were phenotypically normal and a decrease in the number of embryos showing a severe hydrocephalus and yolk phenotype (Fig. 2j). These results suggest that the *flcn* morpholino is specifically targeting the *flcn* transcripts and that flcn may be required for the development of the zebrafish embryo particularly in the growing brain.

### flcn morphants do not show kidney defects or defects in motile cilia in early development

As BHD patients have an increased risk of developing renal cysts and tumours [2] we decided to study the effect of *flcn* knockdown on kidney development in zebrafish embryos. The Wilms Tumor-1b GFP transgenic line (*Tg(wt1b:EGFP))* expresses GFP exclusively in the pronephros and exocrine pancreas [21]. Injection of *flcn* morpholino into *Tg(wt1b:EGFP)* did not reveal any obvious defects in pronephric development at Long-pec stage (Additional file 1: Figure S1H) when compared with mismatch control injected (Additional file 1: Figure S1G)

Morpholino knockdown of *flcn* in zebrafish resulted in a phenotype reminiscent of ciliary gene mutants [22] and morphants [23]. In addition, BHD in humans was recently described as a novel ciliopathy [7]. However, no clear difference in acetylated alpha tubulin staining (a known ciliary marker) in the pronephros and central canal cilia was observed at this magnification in Prim-5 stage embryos injected with *flcn* morpholino (Additional file 1: Figure S1J) when compared with mismatch control injected embryos (Additional file 1: Figure S1I).

### Cell cycle defects in brain development in flcn morphant embryos

The lack of obvious defects in motile cilia morphology and a lack of defects in the developing kidney prompted us to examine other cellular processes that might be affected by Flcn deficiency, thereby causing the morphant phenotype. *In vitro*, FLCN has been implicated in cell cycle regulation via late S and G2/M phase and cyclin D1 [12, 24, 25]. In addition, the *flcn* morphants show developmental arrest and *flcn* is expressed in the proliferative zone of the brain. Therefore, we decided to examine cell cycle regulation in more detail in these zebrafish embryos.

**Fig 3:**
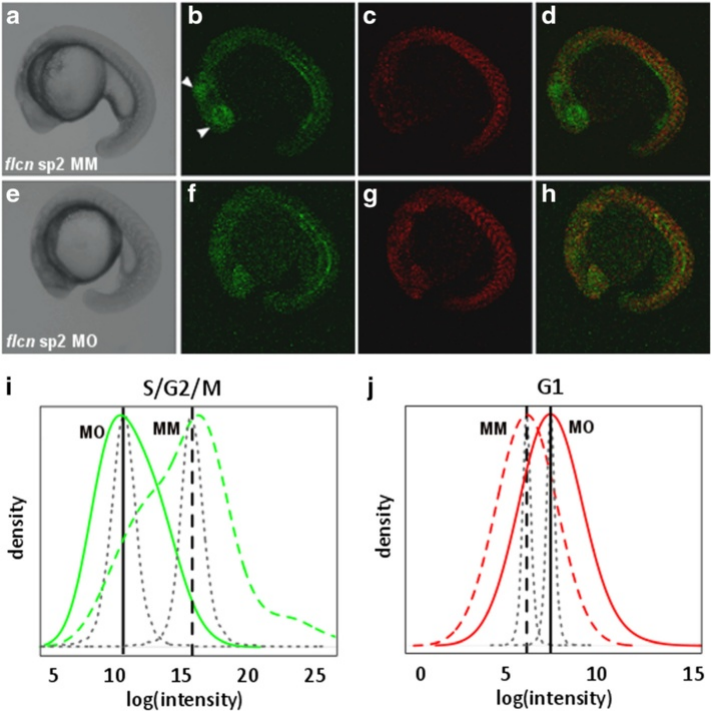
Cell cycle defects in *flcn* morphant embryos at 18 somite stage of development. Zebrafish Fucci embryo injected with *flcn* sp2 mismatch control morpholino imaged in (**a**) Brightfield, (**b**) fluorescence for Geminin-Azami Green (S/G2/M phase), (**c**) fluorescence for Cdt1-Kusabira Orange2 and (**d**) fluorescence merge of Geminin-Azami Green (S/G2/M phase) and Cdt1- Kusabira Orange2 (G1). Zebrafish Fucci embryo injected with *flcn* sp2 morpholino imaged in (**e**) Brightfield, (**f**) fluorescence for Geminin-Azami Green (S/G2/M phase), (**g**) fluorescence for Cdt1-Kusabira Orange2 and (**h**) fluorescence merge of Geminin-Azami Green (S/G2/M phase) and Cdt1- Kusabira Orange2 (G1). **i** Pixel intensity of Geminin-Azami Green in embryos injected with *flcn* sp2 mopholino injected embryos (solid green line (MO)) when compared with *flcn* sp2 mismatch control morpholino injected embryos (*dashed green line* (MM)). Green lines show empirical log(intensity) data distributions. **j** Pixel intensity of Cdt1-Kusabira Orange2 in embryos injected with *flcn* sp2 mopholino injected embryos (solid red line (MO)) when compared with *flcn* sp2 mismatch control morpholino injected embryos (*dashed red line* (MM)). Red lines show empirical log(intensity) data distributions. Vertical, black lines indicate means, with parameter confidence distributions shown as curved black lines. Vertical, black lines indicate means, with parameter confidence distributions shown as curved black lines

To this end we exploited a novel, powerful in vivo labelling tool to monitor cell cycle regulation in zebrafish embryos after *flcn* knockdown. The zFucci system [26] is composed of two transgenes that fluorescently label G1 (red) and S-M phase (green) nuclei in the living zebrafish embryo at remarkable spatial and temporal resolution. The system is based on the detection of ubiquitination of fluorescent fusion proteins Cdt1 with monomeric Kusabira Orange2 and Geminin with monomeric Azami Green. zFucci double transgenic embryos were injected with *flcn* morpholino or mismatch control morpholino and reporter activities indicative of S-M (green) and G1 (red) phases were monitored. Snapshots of green and red fluorescence were used to evaluate the status of cell cycles at approximately 18 somite stage. This analysis indicated a significant drop in the number of cells in S-M phases (Fig. 3i) in *flcn* morpholino injected embryos (Fig. 3e, f, h) particularly in the retina and various compartments of the brain including the tectum when compared with mismatch control morpholino injected embryos (Fig. 3a, b, d). A corresponding increase in G1 cells (Fig. 3j) in *flcn* morpholino injected embryos (Fig. 3g) was seen when compared with mismatch control morpholino injected embryos (Fig. 3c). This argues against general cell death as the reason for decreased detection of S-M phase cells and suggests instead a disruption of the cell cycle in the brain as a result of Flcn knockdown.

**Fig. 4:**
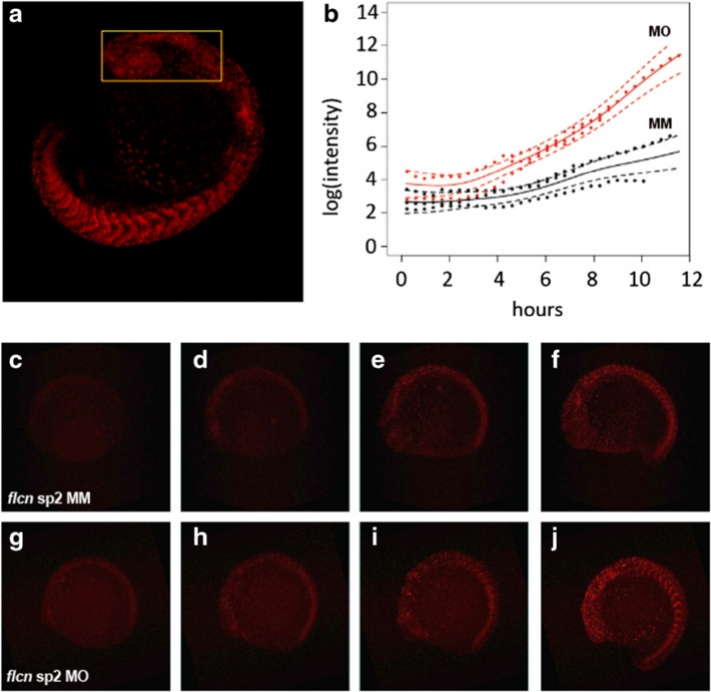
Cell cycle defects in *flcn* morphant embryos during somitogenesis. **a** Zebrafish Fucci embryo showing fluorescence for Cdt1-Kusabira Orange2 (G1 phase) with analysed region of the head indicated by yellow box. **b** Longitudinal time courses of log(intensity) of pixels from 3 *flcn* splice 2 mismatch morpholino and 4 *flcn* splice 2 morpholino injected embryos. Frames were taken at 22 min and 36 s intervals from bud stage for *flcn* splice 2 mismatch morpholino and 2-somite stage for *flcn* splice 2 morpholino injected embryos. Red data points represent *flcn* sp2 morpholino (MO) injected embryos; black data points represent mismatch control morpholino (MM) injected embryos. Solid lines indicate estimated mean trajectories for each treatment group, with dashed lines denoting parameter 95 % confidence intervals. **c** Bud stage, (**d**) 2-somite stage, (**e**) 4-somite stage, (**f**) 10-somite stage *flcn* splice 2 mismatch control morpholino injected embryos. **g** 2-somite stage, (**h**) 4-somite stage, **i** 6-somite stage, (**j**) 10-somite stage *flcn* splice 2 morpholino injected embryos

Next we asked, whether the change in cell cycle behaviour can be attributed to a specific stage of development. To this end, time-lapse analysis of the head region (Fig. 4a*yellow box*) in *flcn* morpholino injected (Fig. 4g, h, i and j) and mismatch control (Fig. 4c, d, e, and f) embryos was carried out over the period from 6 somites to Prim-20 stages by time lapse confocal microscopy on immobilised embryos. The time-lapse analyses show that initially embryos carry comparable levels of cells in G1 phase (Fig. 4b). As time progresses the rates of zFucci transgene activities diverge suggesting divergence in the number of S-M and G1 cells respectively (Fig. 4b) and a gradual increase in cells in G1 phase in *flcn* morpholino injected embryos when compared to mismatch control injected embryos (Fig. 4b). These results suggest that there is no specific stage with dramatic change in cell cycle behaviour, but rather indicates a gradual loss of proliferation of the cells monitored by the zFucci system.

## Discussion

In this study we show the result of gene expression analysis and morpholino knockdown of *flcn* during zebrafish early development. Our results suggest that *flcn* may be required for zebrafish embryonic brain development. Interestingly Birt-Hogg-Dubé (BHD) patients shown no defects in the brain however all known BHD patients only show a mutation in one copy of the *FLCN* gene [6]. Canine and murine animal models homozygous for *FLCN* knockout show early embryonic lethality [27, 28]. Chen et al. and Hasumi et al. showed that mice homozygous for *flcn* mutation die between 3.5 dpc and 8.5 dpc [9] (E5.5–E6.5 [18]). Before death mutant mice lacked an organised epiblast and surrounding visceral endoderm. In embryos that did develop visceral endoderm and ectoderm, these layers had a disorganised structure containing cells with an enlarged cytoplasm and disorientated nuclei. In contrast *flcn* morpholino injected zebrafish, appear to develop normally past gastrulation with the first signs of disruption occurring when the embryos arrest at 18hpf. By Prim-5 stage the embryos exhibit cell death in the brain and trunk and by Long-pec stage the embryos show brain oedema and problems with tail circulation as well as a larger, more fragile yolk and thinner yolk extension.

The morpholino phenotype is supported by whole mount *in situ* hybridization of *flcn* showing expression in the tectum and telencephalon in zebrafish. In mice, *flcn* expression can be seen in the neural tube [18]. At E7.5 *Flcn* showed a strong signal in the neural ectoderm of the primitive streak region and in the headfold of the ectoderm in mice. At E9.5, *Flcn* was expressed almost ubiquitously with a strong signal in the neural tube and optic pit. However, at E10.5 *Flcn* was highly expressed in the branchial arch, forelimb, and hind limb, as well as in the somites. Adult mice showed expression in the heart, pancreas and prostate with moderate expression in adult brain, kidney, liver and lung [18]. These results appear consistent with what we see in zebrafish development where expression of *flcn* is also seen in the fin bud, somites, eye and regions of the brain. This suggests a critical role for *flcn* in vertebrate development.

In humans BHD has recently been described as a novel ciliopathy with FLCN being shown to localise to motile and non-motile cilia, centrosomes and the mitotic spindle. Alteration of FLCN levels caused changes to the onset of ciliogenesis [7]. In zebrafish embryos lacking genes important for ciliogenesis one of the reported phenotypes is hydrocephalus [29], also seen in *flcn* morphants [30]. It therefore seemed probable that knockdown of *flcn* in zebrafish embryos would result in defects in the pronephros and/or other ciliated structures. However we have found no evidence for this when we compare the pronephros of *Tg(wt1b:EGFP)* zebrafish embryos with and without *flcn* knockdown or when using immunofluorescent staining for acetylated alpha tubulin (a ciliary marker). It is possible that although cilia seem to be present in the *flcn* knockdown embryos, they may not be functional. Alternatively, mammalian FLCN is present in both motile and non-motile cilia [7]. In this study we primarily focused on the effect of *flcn* morpholino knockdown on the appearance of motile cilia, as it has been shown that defects in these cilia can cause hydrocephalus and kidney cysts [23, 31, 32]. However aspects of the *flcn* morpholino phenotype such as hydrocephalus and cell cycle defects could also be explained by defects in the non-motile primary cilia, [33, 34] providing interesting avenues for further investigations. Alternatively, FLCN’s function in ciliogenesis may be specific to mammals.

Given the lack of obvious ciliary defects we sought to understand the developmental arrest phenotype. In particular whether this phenotype was caused by defects in the cell cycle as a recent study by Laviolette et al. (2013) suggested that *flcn* delays cell cycle progression through late S and G2/M phase [25]. To do this we knocked down *flcn* in zFucci transgenic embryos where the G1 phase of the cell cycle is labelled red and S, G2 and M are labelled green. We found that at the 18 somite stage there was a significant drop in S-M states (Fig 3j) and a corresponding increase in G1 cells in *flcn* morpholino injected embryos (Fig 3e, f, h). This was particularly evident in the brain, retina and somites of the embryo, areas that have been shown in both mice [18] and zebrafish (Fig 1) to express *flcn*. To investigate at which developmental stage this divergence of zFucci transgene activities arises we undertook timelapse analysis of the head region of *flcn* morpholino injected and mismatch control embryos which show a gradual and increasing change over time. From this we can conclude that Flcn may play a role in the cell cycle. However, the exact nature of Flcn's control on the cell cycle remains to be determined, as recent in vitro studies have suggested that FLCN delays cell cycle progression through late S and G2/M phase [25] and that FLCN regulates cyclin D1 expression [24] which promotes G1 to S phase progression in the cell cycle [35]. Kawai et al. found that RNAi knockdown of FLCN in HeLa cells led to increased cyclin D1 expression and, conversely, that reintroduction of the *FLCN* gene into *FLCN*-null Nihon rat kidney tumour cells reduced cyclin D1 expression. They surmised that these results suggested that FLCN inhibits cyclin D1 expression and, by extension, may also regulate G1 to S phase transition of the cell cycle. Conversely in zebrafish we found that knockdown of *flcn* resulted in no significant change in cyclin D1 (data not shown) suggesting that cell cycle defects in the developing embryo as a whole are likely to be more complex.

Alternatively, the cell cycle defects might be secondary to other consequences of Flcn knockdown. Cells in G1 can only progress if sufficient growth factors such as nutrient supply are present (reviewed [36]). In vitro, FLCN has been shown to regulate signalling of AMPK, a major sensor of energy status. FLCN directly interacts with AMPK [8] and loss of FLCN was shown to constitutively activate AMPK leading to mitochondrial biogenesis and HIF-driven aerobic glycolysis [17, 37]. Lui et al. (2013) have shown that *Drosophila melanogaster* with a genomic deletion of the Flcn gene never survive to adulthood and show characteristics of malnutrition [38]. Zebrafish *flcn* morpholino injected embryos show large, fragile yolks and thin yolk extensions indicative of defects in nutrient supply and digestive problems [39–41]. It is therefore possible that the phenotype we see is a result of the zebrafish being unable to properly utilise its nutritional reserves.

Recent publications have shown that morpholino analysis may often not be corroborated by genetic mutants generated by reverse genetic approaches [42, 43]. The reason for this is unclear and has been suggested to be due to off target effects of the morpholino or hypomorphic alleles in the mutants. However new data by Rossi et al. may shed some light on this [44]. Rossi et al. generated mutations in the zebrafish *egfl7* gene, which showed no obvious phenotype. This was in contradiction to the severe vascular defects seen in wild type embryos injected with an *egfl7* morpholino. After comparing the proteome and transcriptome of these mutant and morphant embryos they found a number of proteins and genes which were upregulated in the mutants but not the morphants. Amongst these were genes that could rescue the morphant phenotype. This could argue for a compensatory network that buffers against deleterious mutations [44].

Morpholino studies – including ours – are supported by controls including mismatch MO injection and mRNA rescue experiments [45]. Since the specific phenotypes we describe are observed by the targeting MO only and the hydrocephalus and yolk phenotypes are rescuable by MO resistant mRNA, it is reasonable to speculate that the observed defects are a combination of a morpholino induced state (such as stress), which generates a yet unexplained embryo environment, in which the targeted *flcn* gene becomes limiting for normal development. In this morpholino injected environment the defects observed are due to the loss of the targeted *flcn* gene. The controls indicate that the defects cannot be attributed to *flcn-*independent off target effects and, therefore, this loss of function approach remains informative about linking *flcn* function to cell cycle regulation during development. Evidence from cell culture experiments suggests hypomorphic effects of FLCN [7], while mouse knockouts die in early development prohibiting meaningful phenotype analyses. Thus our morpholino knockdown study provides an informative starting point to unravel the function of *flcn* in a whole organism vertebrate system. How exactly *flcn* affects development and the cell cycle will be answered by the future generation of flcn mutant embryos. As *flcn* is known to have multiple interacting proteins [8, 46–51], we can not rule out that *flcn* mutants may employ a compensatory network to buffer against loss of *flcn*, a result that would also provide exciting new avenues of investigation.

## Conclusions

In conclusion we show that zebrafish *flcn* is expressed during embryonic development with elevated expression levels in proliferating tissues of the zebrafish embryo. Using *flcn* knockdown coupled with a whole organism based *in vivo* cell proliferation assay we show that *flcn* is likely required for embryonic brain development and that it affects the normal progression of the cell cycle particularly in the growing brain and suggest a previously undescribed role for *flcn* in vertebrates.

## Methods

### Zebrafish husbandry and embryo generation

Zebrafish embryos were obtained from sibling crosses from adult AB fish housed at the fish facility at Birmingham University. Zebrafish were raised and bred and embryos staged following standard protocols [52, 53]. Embryos are raised at 28.5 °C. When needed to prevent pigment formation, embryos were raised in 0.003 % phenylthiocarbamide in E3 medium (1 mM NaCl, 0.17 mM KCl, 0.33 mM MgSO_4_.7H_2_O, 0.33 mM CaCl_2_.2H_2_O) from tailbud stage.

### flcn cDNA cloning and mRNA production

The full open reading frame for zebrafish *flcn* was cloned from total RNA extracted from pooled protruding-mouth stage AB* wild type embryos using TRIZOL reagent according to manufacturers’ instructions. Next, cDNA was produced using M-MLV Reverse Transcriptase, RNase H Minus, Point Mutant (Promega M3681, M3682, M3683) and oligodT primers (Invitrogen). Zebrafish *flcn* cDNA was amplified using the following forward primer with BamHI restriction site: AATA GGATCC ATGAACGCTTTAGTTGCCCTG and reverse primer with Xba1 restriction site: AATA TCTAGA CCCGCTTTCAGTCTCTCTCAC and cloned into pCS2+ using BamHI (New England Biolabs) and XbaI (New England Biolabs). Plasmid was verified by sequence analysis.

Capped RNA was synthesised using 5 μg (5 μl) of NotI linearised *flcn* DNA using SP6 mMESSAGE mMACHINE kit (Ambion). The RNA was cleaned using GenElute™ Mammalian Total RNA Miniprep Kit (Sigma RTN70) as per manufacturer’s instructions Appendix 2.

### Microinjection of embryos with RNA and morpholino

Morpholino Oligonucleotides (GeneTools) sequences are as follows:

- FLCN ATG MO AGGGCAACTAAAGCGTTCATCTGTG
- FLCN splice1 MO ATGACACTCCCCTCTCGCTCACCTC
- FLCN splice1 MM ATcACAgTCCCCTgTCGCTgACgTC
- FLCN splice2 MO CGTTCATCTGGAGGAAACAAACATA
- FLCN splice2 MM CGTTCtTgTGcAGGAtACAAAgATA

Morpholinos were diluted in MO buffer (5 mg/ml phenol red (Sigma), 4 mM HEPES pH 7.2 (Sigma), 160 mM KCl (Sigma)) and 1.4 nl of MO solution was injected into the yolk of the 1 cell stage embryo. RNA was diluted to 100-300 pg/μl in nuclease free water and 1.4 nl was injected into the cell of the 1 cell stage embryo. Embryos were stage matched, anaesthetised using 0.016 % MESAB in E3 embryo medium and imaged with a Nikon SMZ800 stereo microscope and Canon Eos 1100D camera and software.

### Quantitative and RT-PCR analysis of splicing morpholino injected embryos

Total RNA was extracted using TRIZOL according to manufactures’ instructions. For RT-PCR RNA was extracted from 50 pooled Long-pec AB* WT embryos either uninjected or injected with either 100 μM control splice 1 mismatch morpholino or 100 μM *flcn* splice 1 morpholino. For qPCR RNA was extracted from 30 pooled Prim5 Nacre WT embryos either uninjected or injected with either 100 μM of control splice 2 mismatch morpholino or 100 μM of *flcn* splice 2 morpholino. Following DNAse treatment using TURBO DNA-free™ Kit (Ambion AM1907), 1 μg total RNA was subjected to RT-PCR using M-MLV Reverse Transcriptase, RNase H Minus, Point Mutant (Promega M3681, M3682, M3683) and random hexamers (Fermentas SO142). Specific PCR was performed on 5 μl cDNA using GoTaq polymerase (Promega M300) with the following primers: Splice 1 forward 5’TCCCATCACATGACACACAA3’, Splice 1 reverse 5’GAGACTGCGCACACATGC3’ Exon 1 forward 5’CCCATCACATGACACACAAGA3’, Exon 1 reverse 5’TGCATGTTCATCAGCCTTCC3’, Exon 2 forward 5’TGTTTTGTACCGAAGCCGTC3’, Exon 2 reverse 5’CTCACACATGTCCGCTCTCT3’, Exon 1 to Intron Forward 5’TTGCCATTGTCAACGCTTTG3’ and Exon 1 to Intron Reverse 5’AACTGCGAGATATCCAGCCA3’ and the following parameters 95 °C for 5 min, (95 °C for 30 s → 58 °C for 30 s → 72 °C for 30 s) x34 and 72 °C for 5 min. qPCR was performed using FastStart Universal SYBR Green Master (Roche) and primers Exon 1 Forward, Exon 1 Reverse, Exon 2 Forward and Exon 2 Reverse above and control gene primers β-Actin F CGAGCTGTCTTCCCATCCA, β-Actin R TCACCAACGTAGCTGTCTTTCTG, *Rpl13*α F TCTGGAGGACTGTAAGAGGTATGC and *Rpl13*α R AGACGCACAATCTTGAGAGCAG [54] on a Stratagene Mx3000P qPCR machine.

### Whole-mount in situ hybridization, double fluorescent whole-mount in situ hybridization and immunohistochemistry

Whole-mount *in situ* hybridization was carried out as described by Thisse and Thisse [55]. The *flcn* gene probe was transcribed directly from cloned cDNA in pCS2+, linearized with NotI (NEB) and transcribed with T7 polymerase (Promega) using Dig labelling mix (Roche). The *pcna* gene probe was transcribed from a plasmid gifted by F. van Eeden, linearized with Not1 (NEB) and transcribed with SP6 (Promega) using Dig labelling mix (Roche). Pre tail bud embryos were fixed in 4 % paraformaldehyde overnight and then manually dechorionated. Embryos older than Prim-5 stage were manually dechorionated and then fixed in 4 % paraformaldehyde overnight at 4 °C. Embryos were cleared in glycerol and imaged with a Nikon SMZ800 stereo microscope and Canon Eos 1100D camera and software and a Zeiss Axioplan compound microscope and Axiovision software.

Double fluorescent whole mount *in situ* hybridization was carried out as described by [56]. The *flcn* gene probe was transcribed as described above and the *pcna* gene probe was transcribed as described above but labelled with DNP labelling kit (Perkin Elmer). *pcna* was developed with anti-DNP-POD (1:300) and revealed with Tyramide-Cy3 at a concentration of 1/25. *flcn* was developed with anti-DIG-POD (1:300) and revealed with Tyramide-FITC at a concentration of 1/150

Whole mount immunofluorescence of acetylated alpha tublin was carried out as follows: embryos were injected with either *flcn* splice 2 morpholino or mismatch morpholino, stage-matched at Prim-5 stage, manually dechorionated and fixed in 4 % formaldehyde in PBS overnight at 4 °C. Embryos were permeabilised using 10 μg/ml proteinase K (Sigma P4850) in PBS containing 0.1 % tween (PBST) for 20 min at room temperature, followed by re-fixation for 20 min. Non-specific binding sites were blocked by incubation in 10 % newborn calf serum in PBS for 1 h at room temperature, followed by overnight incubation at 4 °C in mouse acetylated alpha tublin antibodies (abcam 24610) diluted 1:200 in block buffer. The following day embryos were washed 4x 30 min in PBST and re-blocked for 1 h. Embryos were incubated in sheep-anti-mouse cy3-conjugated antibodies (Sigma C2181) diluted 1:200 in block buffer overnight at 4 °C, followed by 4x 30 min wash in PBST. Embryos were refixed in 4 % formaldehyde in PBS for 20 min at room temperature. Embryos were mounted in Vectashield (Vector labs) and images captured using a Leica LSI TCS Zoom confocal and Leica software. Images were processed using ImageJ software (NIH).

### Acridine orange staining

Twenty four hour post fertilisation Nacre embryos were incubated in 1 μg/ml Acridine Orange (Sigma A6014) in E3 for 30 min. Embryos were washed, anaesthetised and imaged under fluorescent light on a Leica MZ10 Stereo microscope with Jenoptik ProgRes camera.

### Embryo preparation and Confocal Imaging

For confocal imaging of zebrafish Fucci embryos [57] were injected with either 100 μM *flcn* splice 2 mismatch morpholino or 100 μM *flcn* splice 2 morpholino. Embryos were maintained at 28 °C in Danieaus solution (58 mM NaCl, 0.7 mM KCl, 0.4 mM MgSO_4_, 0.6 mM Ca(NO_3_)_2_, 5.0 mM HEPES pH 7.6) until bud-stage. A 96-well plate (Greiner Bio-One) was prepared by adding 50 μl of 1 % agarose (Bioline, cat. no. BIO-41025) in Danieaus solution covered by 30 μl of ethanol (VWR) to each well and inserting a brass 96 pin spotter (diameter 0.7 mm) into the plate until the agarose set when the spotter was removed and the plate washed thoroughly with distilled water. Embryos were dechorinated on 1 % agarose in Danieaus solution and transferred with 60 μl of Danieaus in to the 96 well agarose plate. Embryos were oriented using a Microlance 3 needle outer diameter 0.51 mm (BD Biosciences). 40 μl of mineral oil (Sigma, cat. no. M5904) was added over the surface of the Danieaus solution. Leica LSI TCS Zoom confocal and software was used to record the time-lapse video. Time-lapse was conducted in xyzt mode using the 532 nm and 488 nm lasers. 40 slices were taken for each stack for 11 h, z volume 358.395 μm lens 5x, optical zoom 1.362 step size 9.19, red gain 1200 offset -4, frame average 4 green gain 1250, offset -5 frame average 8. Frames were taken at 22 min 36 s intervals from bud stage for *flcn* splice 2 mismatch morpholino and 2-somite stage for *flcn* splice 2 morpholino injected embryos. For analysis maximum projection images were prepared, pixel intensities were manually identified in the selected area for all embryos. For still imaging, 3 slices were taken for each image with a frame average 3, 2x lens, z volume 500 μm maximum projections were produced and analysed.

### Confocal imaging of Tg(wt1b:EGFP)

*Tg(wt1b:EGFP)* embryos injected with *flcn* splice 2 morpholino or *flcn* splice 2 mismatch morpholino were stage matched anaesthetised and imaged on a Leica TCS LSI confocal. Images were processed using ImageJ.

### Statistics

#### Reporter activity

Analysis of zFucci reporter activity was carried out by statistical evaluation of pixel intensities of fluorescence signals detected for red and green reporters in *flcn* splice 2 morpholino injected embryos, compared to mismatch controls where a mean of 25 embryos were analysed from each set of injections for 4 independent sets of injections. Mean log pixel intensity was calculated for each imaged embryo. This was performed for red and green channels separately. We tested the hypothesis of differential intensity associated with either *flcn* MO or control MO using a linear mixed-effects modelling framework [58]. Morpholino treatment was fitted as a categorical fixed effect and other sources of experimental variation (day, parental fish) were modelled as multilevel random effects. Treatment contrasts were tested using t-tests and results were validated by generating parameter confidence distributions using MCMC with 10^4^ iterations. (R package: lme4. [59])

#### Time course analysis

Log-transformed intensity data from 3 *flcn* mismatch MO injected and 4 *flcn* splice 2 MO injected embryos was modelled using Generalised Additive Models, within a mixed-effects modelling framework. Cubic smoothing splines were used to model mean trajectories for each morpholino treatment. Following initial visual inspection, biological variation was modelled with separate parameters for each morpholino. A first order autoregressive process was used to model temporal autocorrelation within each time course. (R package: mgcv. [60])

#### Rescue experiment

Following replicate (*n* = 3, mean number embryos per group = 80) sets of injections with different RNA concentrations; phenotypes were categorised as ‘severe’ or ‘mild’ hydrocephalus, or ‘normal’. The proportion of the population showing each of these phenotypes with increasing inject RNA concentration was modelling using ordinal logistic regression (R package: MASS. [61]).

#### qRT-PCR analysis

Quantitative PCR data from morpholino-injected embryos were normalized against mean expression of two housekeeping genes (β-actin and *Rpl13*α [54]). Normalized expression data were analysed using a linear mixed effects model. Morpholino (morpholino, MO, *vs*. mismatch, MM) and exon (1 versus 2) were fitted as interacting fixed effects; with technical replication (*n* = 3), explicitly nested within biological replication (*n* = 3), fitted as random effects. Fixed effects were assessed using likelihood ratio tests. Model residuals were found to meet with assumptions of Normality and homogeneity of variance..

All statistical analysis was performed using R, version 3.02 [62]

## Animal ethics statement

Animals were raised as per Home Office guidelines and all experiments were in accordance with Home Office Animals (Scientific Procedures) Act 1986.

## Additional files

Additional file 1: Figure S1. Knockdown of *flcn* and its effect on cilia and kidney development in Long-pec stage embryos. (A) Electrophoresis gel comparing the size of an amplified *flcn* transcript in wild type (WT) embryos, embryos injected with *flcn* splice 1 mismatch morpholino (sp1 MM) and embryos injected with *flcn* splice 1 morpholino (sp1 MO). (B) Comparative alignment of human *flcn* and zebrafish *flcn* protein sequence using Clustal Omega. The red box indicates the sequence and ensuing stop codon that will be translated when the intron is retained in embryos injected with *flcn* sp1 MO. (*) indicates positions which have a single, fully conserved residue. (C) Long-pec stage embryo injected with 100 μM splice 1 mismatch morpholino (D) Long-pec stage embryo injected with 100 μM splice 1 morpholino. (E) Long-pec stage 600 μM ATG control morpholino injected embryos. (F) Long-pec stage 600 μM *flcn* ATG morpholino injected embryo. (G) Fluorescent imaging of *Tg(wt1b:EGFP)* transgenic zebrafish embryos injected with 100 μM splice 2 mismatch morpholino. (H) Fluorescent imaging of *Tg(wt1b:EGFP)* transgenic zebrafish embryos injected with 100 μM *flcn* splice 2 morpholino (*n* = 8, 2 independent experiments). Images are of the dorsal aspect behind the head region with the head positioned to the left. gl, glomerulus; pt, pronephric tubule (I) Acetylated alpha tubulin-cy3 staining of cilia in 100 μM splice 2 mismatch morpholino injected Prim-5 stage embryos (J) Acetylated alpha tubulin antibody staining of cilia in 100 μM splice 2 morpholino injected Prim-5 stage embryos (*n* = 10, 2 independent experiments). Images are of the side view of the trunk of the embryo. cc, central canal; pd, pronephric duct. (TIF 13750 kb) Additional file 2: Table S1. Absolute numbers of embryos after injection with control, mismatch or *flcn* morpholinos. Columns show: injection group - where each group was injected on a different day, treatment group – where embryos were either uninjected, injected with control MO, *flcn* ATG MO, *flcn* sp1 MO, *flcn* sp1 MM, *flcn* sp2 MO or *flcn* sp2 MM, classification of embryos – showing the number of embryos which are dead, normal or abnormal. (XLSX 8 kb)

## Abbreviations

dpf: days post fertilization
hpf: hour post fertilization
LOF: loss of function
MM: mismatch
MO: morpholino antisense oligonucleotides
