# Supplementary figures and images for: Expression and knockdown of zebrafish folliculin suggests requirement for embryonic brain morphogenesis
Source: BMC Dev Biol. 2016 Jul 8;16:23. doi: 10.1186/s12861-016-0119-8 (PMC4939010; doi:10.1186/s12861-016-0119-8)

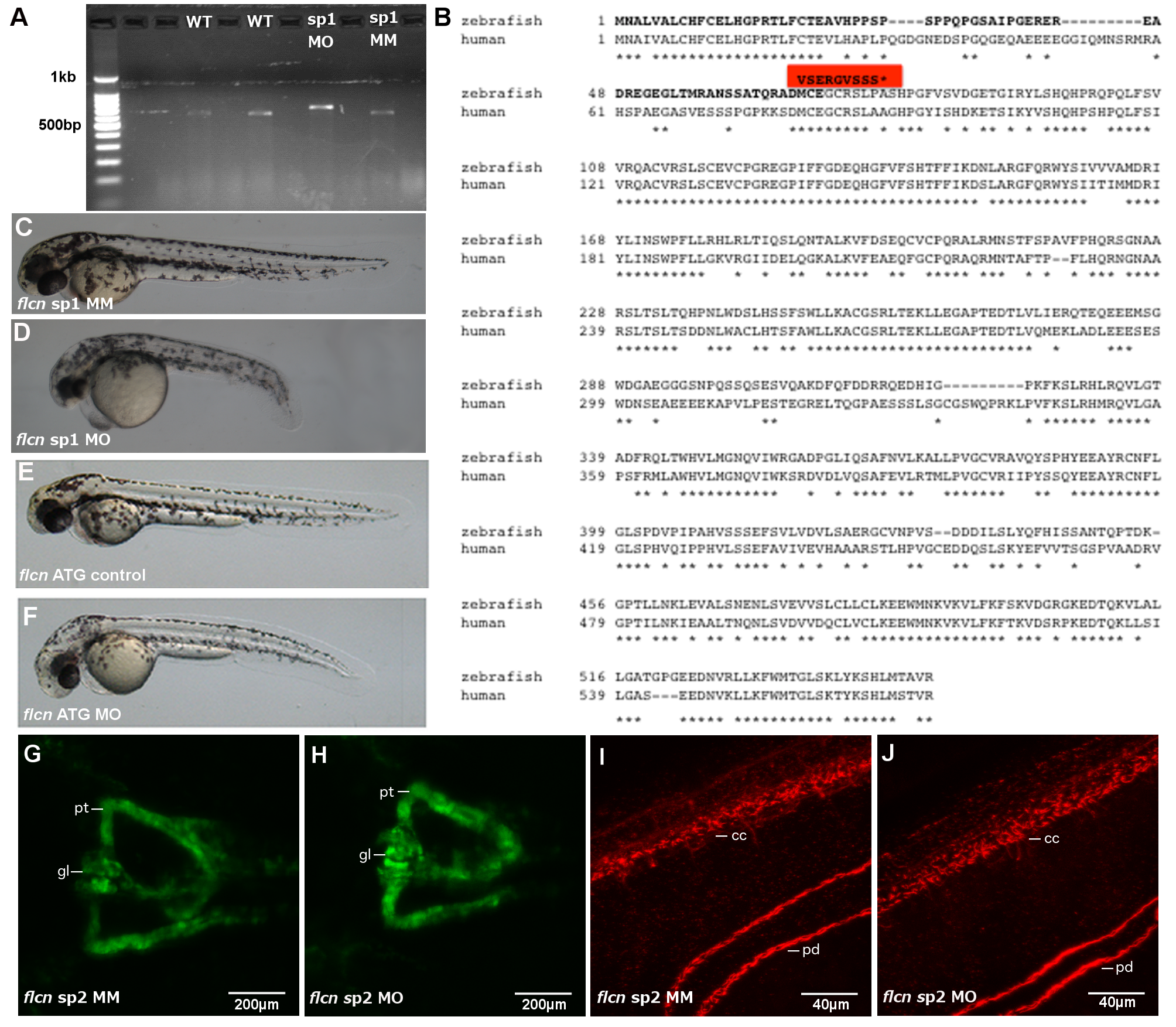

Supplement: Additional file 1: Figure S1. — Knockdown of flcn and its effect on cilia and kidney development in Long-pec stage embryos. (A) Electrophoresis gel comparing the size of an amplified flcn transcript in wild type (WT) embryos, embryos injected with flcn splice 1 mismatch morpholino (sp1 MM) and embryos injected with flcn splice 1 morpholino (sp1 MO). (B) Comparative alignment of human flcn and zebrafish flcn protein sequence using Clustal Omega. The red box indicates the sequence and ensuing stop codon that will be translated when the intron is retained in embryos injected with flcn sp1 MO. (*) indicates positions which have a single, fully conserved residue. (C) Long-pec stage embryo injected with 100 μM splice 1 mismatch morpholino (D) Long-pec stage embryo injected with 100 μM splice 1 morpholino. (E) Long-pec stage 600 μM ATG control morpholino injected embryos. (F) Long-pec stage 600 μM flcn ATG morpholino injected embryo. (G) Fluorescent imaging of Tg(wt1b:EGFP) transgenic zebrafish embryos injected with 100 μM splice 2 mismatch morpholino. (H) Fluorescent imaging of Tg(wt1b:EGFP) transgenic zebrafish embryos injected with 100 μM flcn splice 2 morpholino (n = 8, 2 independent experiments). Images are of the dorsal aspect behind the head region with the head positioned to the left. gl, glomerulus; pt, pronephric tubule (I) Acetylated alpha tubulin-cy3 staining of cilia in 100 μM splice 2 mismatch morpholino injected Prim-5 stage embryos (J) Acetylated alpha tubulin antibody staining of cilia in 100 μM splice 2 morpholino injected Prim-5 stage embryos (n = 10, 2 independent experiments). Images are of the side view of the trunk of the embryo. cc, central canal; pd, pronephric duct. (TIF 13750 kb) [file 12861_2016_119_MOESM1_ESM.tif]
